# Water as an Intrinsic Structural Element in Cellulose Fibril Aggregates

**DOI:** 10.1021/acs.jpclett.2c00781

**Published:** 2022-06-09

**Authors:** Pan Chen, Jakob Wohlert, Lars Berglund, István Furó

**Affiliations:** † Beijing Engineering Research Centre of Cellulose and Its Derivatives, School of Materials Science and Engineering, Beijing Institute of Technology, 100081 Beijing, P.R. China; ^‡^Department of Fiber and Polymer Technology, ^§^Wallenberg Wood Science Center, and ^∥^Department of Chemistry, KTH Royal Institute of Technology, SE-10044 Stockholm, Sweden

## Abstract

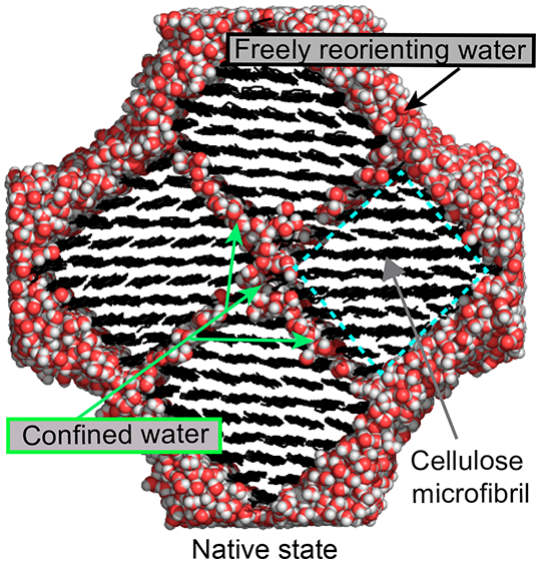

While strong water association with cellulose in plant cell walls and man-made materials is well-established, its molecular scale aspects are not fully understood. The thermodynamic consequences of having water molecules located at the microfibril–microfibril interfaces in cellulose fibril aggregates are therefore analyzed by molecular dynamics simulations. We find that a thin layer of water molecules at those interfaces can be in a state of thermal equilibrium with water surrounding the fibril aggregates because such an arrangement lowers the free energy of the total system. The main reason is enthalpic: water at the microfibril–microfibril interfaces enables the cellulose surface hydroxyls to experience a more favorable electrostatic environment. This enthalpic gain overcomes the entropic penalty from strong immobilization of water molecules. Hence, those particular water molecules stabilize the cellulose fibril aggregates, akin to the role of water in some proteins. Structural and functional hypotheses related to this finding are presented.

Water is one key factor for processing–structure–property relationships of cellulose nanomaterials. It influences chemical functionalization of cellulose surfaces, as well as cellulose–polymer interfacial adhesion in polymer matrix nanocomposites. In its natural form, cellulose is present as “microfibrils” or “elementary fibrils”, that is, ordered assemblies of its polymeric chains in extended conformation. In man-made materials, the term cellulose nanofibrils is also often used to describe the same object. Here, the term “microfibril” is employed for both fibrils in plant cell walls and fibrils disintegrated for use in man-made materials. They have lengths in the order of micrometers and widths that range from a few nanometers in plants to several tens of nanometers in other organisms.^1−6^ In the plant cell wall, the microfibrils often form larger aggregated structures: fibril aggregates (FA, also called microfibrillar bundles).^1,3,5,7^ The aggregate structure is certainly present not only in primary and secondary^8^ cell walls but also in man-made, chemically processed wood fibers.^9^ These aggregates become tightly bound after drying and resist both swelling and high levels of mechanical shear, and their formation is possibly predetermined by geometrical constraints directly upon biosynthesis.^1,10^ Yet, microfibrils can be disintegrated from the FAs and individually dispersed in water.^11^

Recently, strong and direct evidence was obtained by ^2^H solid-state NMR for the existence of two separate classes of water molecules in cellulose hydrated at different relative humidities (RH, in the whole range of 33–93%).^12^ Previous ^1^H NMR studies suggested this possibility^13,14^ but were hampered by the difficulty of separating complex signal contributions from water and exchangeable and nonexchangeable carbohydrate protons. Different types of water have also been part of speculations about mechanisms in water sorption studies,^15−17^ including kinetic ones.^18^ Scattering experiments have also hinted at such possibilities.^19−21^ It is important to clarify that the NMR findings^12^ were obtained up to approximately 20 wt % of water content where all adsorbed water behaves as “non-freezing”,^22^ that is, showing no bulk-like phase transition. This “non-freezing” behavior is a general and long-established feature^23^ in all biomolecular systems with water adsorbed at content below “usually in the range 0.4 to 1.0 grams of water per gram of macromolecule”, the exact value depending on the actual biomolecule or biomolecular assembly. Thereby the two classes of water discussed here should *not in any way* be identified as “freezing” and “non-freezing”.

One of the classes of water identified by NMR^12^ showed a behavior with *one* similarity to bulk water: namely, the molecules exhibited *isotropic* molecular reorientations (and therefore, as in bulk water, yielded an ^2^H NMR peak without static quadrupole splitting), albeit much slower. This fraction increased strongly with increasing RH and represented most of the water at high RH. Considering the swelling behavior of cellulose, this class was assigned to water molecules residing *among* the fibril aggregates. The second class of water contained molecules of highly specific and unusual behavior.^12^ Molecular motions were slow and strongly *anisotropic* (as witnessed by its ^2^H NMR spectrum with large quadrupole splitting), a feature indicating strong binding to cellulose microfibrils. The fraction of water molecules in this second class leveled off upon increasing RH, and only a minor fraction was present at high humidity. In addition, water molecules showed slow interchange between the two classes. This last feature suggested a distinct and extended spatial location for the water molecules in the second class. The only plausible candidate location was the disordered interface between the microfibrils *within* the fibril aggregates. Hence, we assume that the microfibril–microfibril interfaces within aggregates to be accessible to water. A fundamental question arises then: can water located within FAs be in a thermodynamically stable state or not, or in other words, can water molecules residing in the interior of the FAs be in thermal equilibrium with the hydrated exterior or not? In the present study, this is investigated using molecular dynamics (MD) simulations, and the answer is, indeed, positive. Moreover, the answer we obtain points to the fundamental role water plays in FAs.

Fibril aggregates (FAs) are constituted by individual microfibrils rather than a single, larger fibril, and thereby a FA has intrinsic microfibril–microfibril interfaces with associated disorder. A question is why the microfibrils are prevented from fusing/coalescing into a larger^24^ crystalline unit. There is currently no consensus^25^ around the answer, but it has been suggested that in the plant cell wall, hemicelluloses may prevent aggregation by sorption to the microfibril surface so that hemicelluloses are trapped between the microfibrils.^3,8,26^ However, considering the approximate dimensions of wood or primary cell wall cellulose microfibrils, there seems to be an insufficient amount of hemicellulose to completely coat cellulose surfaces. A hemicellulose content of 25% results in 1 hemicellulose macromolecule per three cellulose macromolecules of equal length, which is just enough to cover the internal surfaces in a FA. Even if hemicelluloses adsorb to cellulose in a tightly packed fashion indicated by recent experimental works,^27,28^ complete coverage is statistically unlikely, and thereby plenty of direct microfibril–microfibril contact remains.^8^ In the context of the simulations below, having a partly hemicellulosic (yet, tightly adsorbed) surface instead of a purely cellulosic one is not a principally different situation. Another possibility, further explored in this work, is that the microfibrils within an FA are dominantly aligned in an antiparallel manner^1,29,30^ that prevents fusion (that is, of microfibrils of cellulose Iβ). In contrast, parallel microfibrils could fuse by a simple spatial shift of them relative to each other. Another possible explanation for limited fibril fusion is that microfibrils show a slight twist^31,32^ in crystalline orientation along the fiber axis, which would impede fusion of axially parallel units. The hypotheses are *not* mutually exclusive, and irrespective of the mechanism, the presence of microfibril–microfibril interfaces *within* the FA is well-established. At such an interface, intermolecular interactions including the number of interchain hydrogen bonds are reduced compared to intermolecular interactions *inside* an individual microfibril. Hence, the presence of microfibril–microfibril interfaces results in a free energy penalty. In light of the long evolutionary history of cellulose and its function in a large variety of biological organisms, we are interested in two additional questions: (i) in which way is the energy/enthalpy penalty (reduced intermolecular interactions) compensated for, and (ii) why are fibril aggregates advantageous for an organism? We will now address the first question and speculate about the second one.

The structural reason(s) for forming disordered cellulosic microfibril–microfibril interfaces are not critical for our discussion below, and findings regarding the role of water remain relevant irrespective of these reason(s). Thus, a minimal FA system is suggested, which is not arbitrary but provides essential structural features with limited variability. It consists of four microfibrils in antiparallel alignment. This comparably simple model still results in disordered interfaces within the FA, and the microfibrils are unable to fuse into a larger crystallite unit. The main features of the simulated system are illustrated in Figure 1. The model FA is the same as that used in a previous study^33^ of molecular motions in cellulose fibril aggregates. It is based on four microfibrils of finite length with a degree of polymerization of 40, aligned in an antiparallel manner. Each microfibril consists of 36 cellulose chains in a six-by-six configuration according to the established crystal structure of cellulose Iβ.^34^ There is growing evidence that the wood microfibril may contain as few as 18–24 chains,^1^ and the actual shape may be a diamond-shaped arrangement of the glucan chains. Because the purpose of the present simulations is not to settle structural issues, a minimal model capturing essential features is used for atomistic simulations (see Figure 1). The distance between cellulose surfaces is initially ∼0.3 nm, and water molecules were randomly inserted at the cellulose microfibril–microfibril interfaces within the FA structure. The FA was also hydrated at its *external* surface by adding water molecules so that a thick solvent layer was obtained. The number of water molecules was selected so that (i) the total water content corresponded to the experimental moisture content at 92% RH (14585 water molecules, or 22 w%, a figure used in our previous simulation^33^) and (ii) roughly 10% (1354) of all water molecules resided at the cellulose microfibril–microfibril interfaces.^12^ The water coverage at the interfaces *within* the FA is such that the number of water molecules per interior surface glucose unit (that is, within the FA) is roughly 0.85; hence, the water is present approximately as a monomolecular layer. This system was then equilibrated for 170 ns, followed by a production phase of 260 ns during which the total system energy was sampled and averaged for every 20 ns data block. Finally, the energy averaged over the blocks with its corresponding error^35^ was calculated.

**Figure 1:**
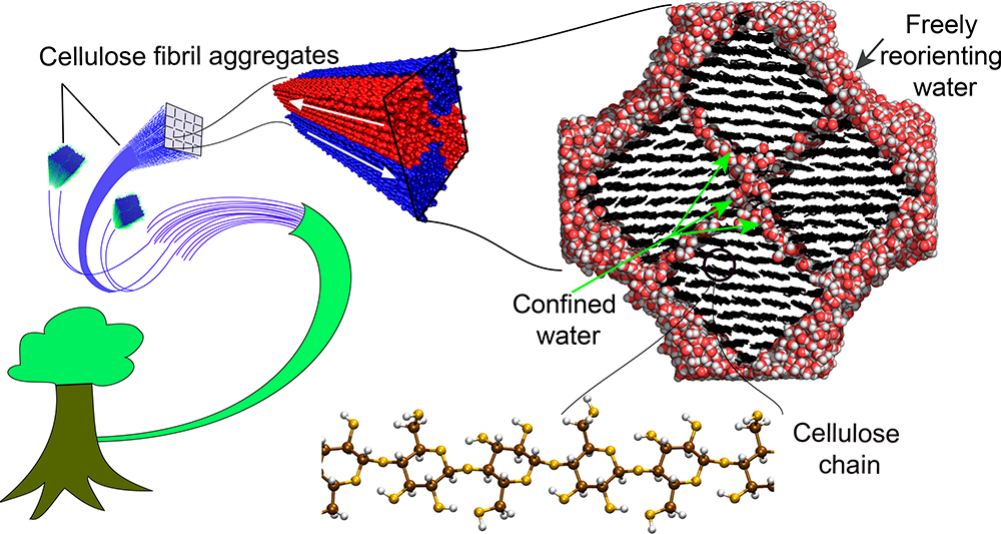
Hierarchical structure of the explored cellulose fibril aggregate (FA) model constituted by four antiparallel microfibrils.

The interfibril water molecules in the FA interior are free to exchange with the external hydration layer. In practice, this process is extremely slow and is not completed on simulation time scales. This is in line with the experimental findings indicating that the lifetime of individual water molecules at the interfaces *within* the FA is in the order (or above) of tens of microseconds.^12^ A central question, which was also touched upon in a previous study^36^ mainly concerned with high-temperature treatment of cellulose, is whether these water molecules are in equilibrium with the external hydration layer or if the system is kinetically trapped in a metastable state.^29^ A recent simulation study^37^ indeed shows that water molecules trapped between two individual microfibrils in solution can give rise to a metastable state. However, the study also indicates that in a FA the situation may be different because of the additional constraints imposed by neighboring microfibrils and possibly twisting, which effectively limits the possibility for interface formation. In thermal equilibrium, the free energy change for transferring a water molecule from the interior of the FA to the external hydration layer is zero.

To test this, more complex simulations using, for example, thermodynamic integration^38^ could be performed to clarify the chemical potential difference between the two different locations. A simpler, yet illustrative and computationally less costly, method was selected here that still estimates free energy change as water is interchanged between the interior cellulose microfibril–microfibril interfaces and the water phase outside the FA, and vice versa. Here, the energy difference relative to a reference state with no interior water was computed by removing all 1354 interior water molecules and adding them to the exterior hydration layer. The two states prepared are illustrated in Figure 2.

**Figure 2:**
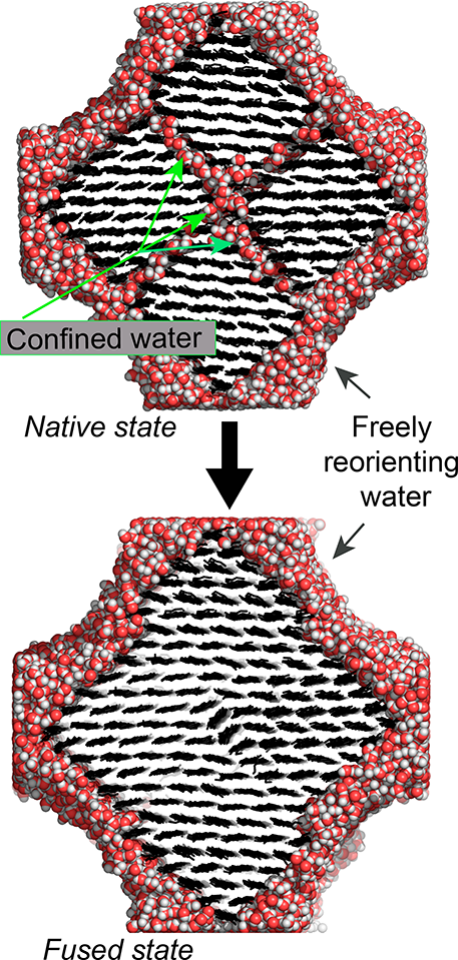
Hydrated (native) state of fibril aggregates with water molecules at the interface between microfibrils and the dry state (fused) where those water molecules were moved to the water phase surrounding the fibril aggregate.

The system was then equilibrated for 170 ns, and the total system energy for the 260 ns production phase was sampled as for the hydrated system. These simulations show that the transfer of water *from* the water phase around the FA *into* the cellulose interfibril region, inside the FA, is exothermic with an average energy change Δ*E* = −7.5 ± 0.2 kJ/mol per water molecule (Table 1). We stress that the equilibration time of 170 ns was sufficient to converge the system to a stable configuration in both its fused dry and hydrated states (see the Supporting Information).

**Table 1:** Decomposition of the Potential Energy Difference (Δ*E* = *E*_hydrated_ – *E*_dry_, in kJ/mol) per Water Molecule between Fibril Aggregates (i) with Native Hydrated Internal Interfaces (*E*_hydrated_) and (ii) with Fused Internal Interfaces Devoid of Water (*E*_dry_)^a^

total Δ*E*	electrostatic	Lennard-Jones	conformational
–7.5	–9.4	4.3	–2.3

a The different terms in the decomposition are the differences between the respective energies for the two states. The electrostatic and Lennard-Jones terms are both the sums of all respective intermolecular terms, while the conformational term summarizes the intramolecular energy (dependent on the variable bond angles) of the cellulose chains.

Although electrostatic interactions are dominating, there is sizable and opposite contribution from dispersion forces, presumably dominating the Lennard-Jones term (see Table 1). Water is known to exhibit weak dispersion forces, and adding water to the internal interfaces weakens the stronger cellulose–cellulose terms. Finally, adding water seems to reduce the conformational strain experienced within the cellulose, and this contributes favorably to the total potential energy through reduced contributions from angles and dihedrals.

The entropy component Δ*S* for this process is more difficult to calculate from simulations. However, it is possible to obtain meaningful estimates from known thermodynamic data.^39^ It is apparent that Δ*S* must be negative and that its magnitude certainly is smaller than the molar entropy of fusion Δ*S*_fusion_ = 22 J/mol·K. The reason is that Δ*S*_fusion_ represents the transfer of a bulk ice water molecule (with *lower* molar entropy than the water at the cellulose microfibril–microfibril interface) to bulk water in the liquid state (with *higher* molar entropy than the adsorbed water outside the FA). Given this highly conservative estimate for Δ*S*, the (Helmholtz) free energy of transfer Δ*F* = Δ*E* – *T*Δ*S<* Δ*E* + *T*Δ*S*_fusion_ ≈ −1 kJ/mol (at ambient *T* = 293 K). Even with this crude overestimate of the entropy term, the average free-energy change per water molecule is *negative*. This important result means that the system with water molecules at the interior fibril interfaces has lower free energy than the reference state, where no water is located between the microfibrils.

An improved, yet conservative estimate can be obtained by considering that the differential (relative to the entropy change at condensation into bulk water) change of entropy upon water adsorption to cellulose is approximately ΔΔ*S*_vapor_ ≈ 14.5 J/mol·K.^40,41^ The entropy for adsorbed water, primarily outside the fiber aggregate, is significantly lower than for bulk water, and a better estimate for Δ*S* is obtained from Δ*S* = −Δ*S*_fusion_ + ΔΔ*S*_vapor_ ≈ −7.5 J/mol·K. The free energy of transfer then becomes Δ*F* ≈ −5 kJ/mol. Hence, the free energy of water molecules in a close-to monolayer arrangement at the cellulose microfibril–microfibril interfaces is significantly lower than for water molecules externally solvating the FA. In conclusion, there is a thermodynamic driving force for the interpenetration of water, which supports the notion that *water can indeed be present at the cellulose microfibril–microfibril interfaces*.

The factors behind the average potential energy difference per water molecule Δ*E* = −7.5 ± 0.2 kJ/mol need to be analyzed further. In Table 1, we also provide the various terms that jointly provide this effect. The main contribution to the energy gain upon inserting water molecules at the internal interfaces is from electrostatic interactions between charge densities located on water and microfibrils. Often, such electrostatic effects are conceptualized as hydrogen bonds.^7^ Indeed, by using the standard criteria of hydrogen bonds (cutoff of donor–acceptor distance less than 0.35 nm and HD···A bond angle less than 30°) we find an additional 188 hydrogen bonds with internal microfibril–microfibril interfaces hydrated (Table 2). In our model and in this context, the cellulose microfibrils within the fibril aggregates are antiparallel, and this structural mismatch will limit the extent of cellulose–cellulose hydrogen bonds at the microfibril–microfibril interface. It will also limit the favorable dispersion interactions that arise from optimized packing, which is the major contribution to the cohesive energy of cellulose.^42^

**Table 2:** Average Number of Various Types of Hydrogen Bonds (HBs) in the Hydrated Fibril Aggregates Model with the Internal Microfibril–Microfibril Interfaces Either Fused and Devoid of Water (“dry”) or Hydrated to the Extent of about One Monomolecular Layer^a^

distance D···A < 0.35 nm and angle HD···A < 30°	dry interface	hydrated interface	difference	net
water–water HBs	20864	19432	–1414	
water–fibril HBs	4306	6784	+2478	188
microfibril–microfibril HBs	14728	13852	–876	

a The hydrated system has 188 more hydrogen bonds in total.

As water is removed from the external hydration layer and added to the interfibril interface region, the number of water–water hydrogen bonds is of course reduced. This, however, is more than compensated for by the increased number of hydrogen bonds *between* water and the cellulose surface hydroxyls groups, which was also noted previously.^37^ Yet, while the electrostatic interaction energy is defined exactly (for selected potentials), the “hydrogen bond” concept remains somewhat arbitrary.

It is important that irrespective of the actual conceptualization of the electrostatic effects, water molecules in the microfibril–microfibril interface region have significantly larger rotational and translational freedom than hydroxyl groups at the cellulose surfaces. For this reason, water and its surface charge distribution can adapt favorably to the charge densities located mainly on surface hydroxyl groups on cellulose microfibrils, which are present at the interfibril interface between two adjacent cellulose surfaces. This geometric freedom is an advantage offered by the small water molecules. Our findings provide, first, support for the proposal that water molecules at the cellulose microfibril–microfibril interfaces can indeed be an *equilibrium* feature. Second, an explanation is given for the experimental observations of the slow and anisotropic molecular reorientation dynamics of some water in cellulose.^12^ Namely, water molecules at the internal interfaces are often bound to/by several immobile cellulose hydroxyls, and for that reason they exhibit only very limited reorientational freedom.

It is important to remember that the calculated free energy of transfer is an average quantity that represents the difference between the specific hydrated state chosen for the simulations and the state with no water at the internal interfaces. We have established that the specific hydrated state is lower in free energy than the nonhydrated state, from which we first of all infer that the true thermodynamic equilibrium should contain water within the cellulose fibril aggregates. Yet, in true thermodynamic equilibrium the chemical potential for water within and outside FAs must be identical. With regard to that, we can provide an additional consideration (that is, beyond the necessarily approximate nature of the applied potentials).

As in the current model, a fibril aggregate exposed to water will gain more internal water molecules and thereby show water-induced swelling. However, this process cannot proceed indefinitely, which means that the transfer free energy per water molecule must level off to zero. Plausibly, there is a quickly diminishing potential energy effect if the cellulose surface hydroxyls at the cellulose microfibril–microfibril interface have all been intermolecularly accommodated by water (or cellulose). If the number of water molecules is increased beyond that point, the free energy gains will come to a halt (because moving water to those interfaces should always lead to some entropic penalty). If so, a limited amount of water, such as approximately a monolayer, may result in a minimum free energy arrangement which permits a maximum extent of electrostatic interactions. Note that the microfibril–microfibril interactions summarized by hydrogen bonds are still the most extensive ones and, henceforth, the FA remains intact.

There are interesting similarities with the behavior of water molecules in a variety of crystalline organic hydrates,^43^ including those based on certain noncellulosic carbohydrates (such as cyclodextrins). In the latter group, the presence of water as a regular stoichiometric structural element which influences the actual molecular arrangement and conformations is well accepted.^44−47^ The presence of strongly bound water molecules in proteins^48^ and nucleic acids^49^ with associated structural and functional roles is well-established, and this is also the case in some synthetic polymer fibers.^50^ Permitting the surface hydroxyls to establish strong hydrogen bonds may also contribute to less disorder in fibril aggregates relative to that in dispersed microfibrils, a feature that is observed in experiments.^51^

Cellulose fibrils have a mechanical load-bearing function as tensile materials in primary and secondary cell walls. In this context, water certainly has strong effects on the mechanical behavior of cellulosic systems.^52,53^ Water facilitates interfibril shear deformation^54−58^ and possibly has a function in cell wall plasticity. The present investigation provides a new perspective regarding the role of water and shows that, in a thermodynamic sense, water may function as an adhesive at the cellulose microfibril–microfibril interface. Water molecules can penetrate deeply into cellulose fibril aggregates where they partly occupy the free volume created by the intrinsic structural disorder at the microfibril–microfibril interfaces. Water then creates a favorable electrostatic environment for the localized charge densities on the microfibril surface. This is accompanied by a net decrease in potential energy, which more than compensates for the loss of rotational and translational entropy for water molecules. This has the net effect of *minimizing* the free energy of the system. In other words, water acts as an adhesive. This finding modifies the long-established opinion supported by many previous investigations, in which water weakens the cohesiveness of cellulosic materials constituted by larger and less regularly arranged structural features (corresponding to our FAs and beyond) of fibrous cellulose. Clearly, wet paper is easy to tear.

The notion of an intrinsic structural role of water molecules in cellulose fibril aggregates is a new result, and this water is likely to contribute toward ductility and plasticity of cells in living plant organisms. Without thermodynamically stable interfacial water molecules, also man-made cellulosic materials would suffer from increased brittleness because this would limit plastic deformation mechanisms at interfibril interfaces. The present findings are important for cellulose nanomaterial investigations, e.g., cellulose surface modification, molecular adsorption studies, and cellulose–polymer interfaces in fibrous reinforcements (plant fibers and nanocelluloses) containing fibril aggregates.

## Computational Methods

The cellulose was modeled using the GLYCAM06 carbohydrate force field,^59^ with the TIP3P potential for water.^60^ Simulations were run with GROMACS 2019^61^ in an *NVT* ensemble (thereby relevant for Helmholtz free energy) using a basic time step of 2 fs. All bonds were constrained to their equilibrium values using LINCS,^62^ nonbonded interactions were cut off at 1.2 nm, and long-range electrostatics was included using particle-mesh Ewald summation (PME)^63,64^ The temperature was maintained at 300 K using stochastic velocity rescaling.^65^

## Supporting Information Available

Calculated energies of the hydrated and the dry system during the production periods (260 ns, subsequent to 170 ns equilibration periods) of the simulation (PDF)
