# Supplementary material for: Water as an Intrinsic Structural Element in Cellulose Fibril Aggregates
Source: J Phys Chem Lett. 2022 Jun 9;13(24):5424–30. doi: 10.1021/acs.jpclett.2c00781 (PMC9234975; doi:10.1021/acs.jpclett.2c00781)
Supplement: Supplementary file 1 — jz2c00781_si_001.pdf [file jz2c00781_si_001.pdf]

Supporting Information

for

Water as an Intrinsic Structural Element in Cellulose Fibril Aggregates

Pan Chen,[1,2,3] Jakob Wohlert,[2,3] Lars Berglund,[2,3] & István Furó[3,4]*

[1] Beijing Engineering Research Centre of Cellulose and Its Derivatives, School of Materials Science and Engineering, Beijing Institute of Technology, 100081, Beijing, P.R. China.

[2] Department of Fiber and Polymer Technology, [3]Wallenberg Wood Science Center and [4]Department of Chemistry, KTH Royal Institute of Technology, SE-10044 Stockholm, Sweden.

*furo@kth.se

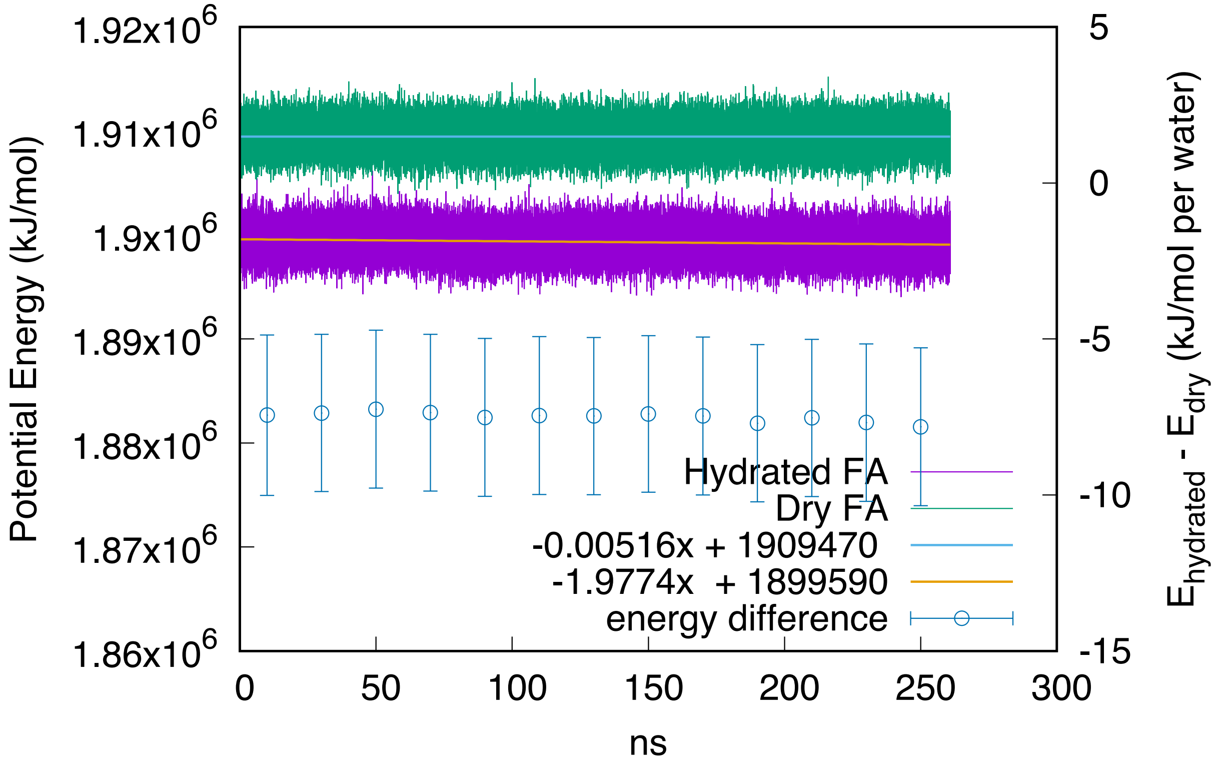

Figure S1. The calculated energies of the hydrated and the dry system during the production periods (260 ns, subsequent to 170 ns equilibration periods) of the simulation, see details in the main text, and the corresponding energy difference calculated from averages for 20 ns data blocks.
